# Evaluation of a genetic risk score computed using human chromosomal-scale length variation to predict breast cancer

**DOI:** 10.1186/s40246-023-00482-8

**Published:** 2023-06-16

**Authors:** Charmeine Ko, James P. Brody

**Affiliations:** Department of Biomedical Engineering, University of California, Irvine, USA

**Keywords:** Copy number variation, Breast cancer, Machine learning, h2o, Germline, UK biobank, TCGA

## Abstract

**Introduction:**

The ability to accurately predict whether a woman will develop breast cancer later in her life, should reduce the number of breast cancer deaths. Different predictive models exist for breast cancer based on family history, BRCA status, and SNP analysis. The best of these models has an accuracy (area under the receiver operating characteristic curve, AUC) of about 0.65. We have developed computational methods to characterize a genome by a small set of numbers that represent the length of segments of the chromosomes, called chromosomal-scale length variation (CSLV).

**Methods:**

We built machine learning models to differentiate between women who had breast cancer and women who did not based on their CSLV characterization. We applied this procedure to two different datasets: the UK Biobank (1534 women with breast cancer and 4391 women who did not) and the Cancer Genome Atlas (TCGA) 874 with breast cancer and 3381 without.

**Results:**

We found a machine learning model that could predict breast cancer with an AUC of 0.836 95% CI (0.830.0.843) in the UK Biobank data. Using a similar approach with the TCGA data, we obtained a model with an AUC of 0.704 95% CI (0.702, 0.706). Variable importance analysis indicated that no single chromosomal region was responsible for significant fraction of the model results.

**Conclusion:**

In this retrospective study, chromosomal-scale length variation could effectively predict whether or not a woman enrolled in the UK Biobank study developed breast cancer.

**Supplementary Information:**

The online version contains supplementary material available at 10.1186/s40246-023-00482-8.

## Introduction

Over 600,000 women die annually from breast cancer around the world [1], but breast cancer is curable (through mastectomy) in the early stages and could be preventable, through prophylactic mastectomy, if one could better predict who will develop breast cancer [2].

Breast cancer predictive models based on genetics already exist. The effectiveness of these predictive models can be characterized by the area under the receiver operating characteristic curve, known as the AUC. One commonly used predictive model, the Gail model [3], has an AUC of 0.58 (95% confidence interval [CI] = 0.56 to 0.60) [4]. The Gail model incorporates a number of parameters including first degree relatives who were diagnosed with breast cancer. The Tyrer-Cuzick model includes a more detailed picture of genetics including BRCA1/BRCA2 status and a hypothetical low-penetrance gene that is designed to encompass all other genetic factors [5]. The Tyrer-Cuzick model is an improvement over the Gail model and has an AUC = 0.62, with a 95% CI of (0.60 to 0.64) [6]. The Tyrer-Cuzick model has been extended using a 313 variant polygenic risk score. This extension improves the AUC to 0.64 with a 955 CI of (0.61 − 0.68) in women over 50 years of age [7].

Family history is encoded in the germline genetics. In fact, models based on detailed germline genetics should perform better than models based on family history alone, since family history is often incomplete; limited to just a generation or two, and genetic factors present in relatives might not be inherited. The more recent approach to predicting breast cancers is to incorporate polygenic risk scores.

Polygenic risk scores, computed from linear combinations of SNPs, should provide superior predictions compared to models that rely on family history questionnaires, but they do not show a substantial improvement. The most complete study to date used a group of 313 SNPs to predict breast cancer with an AUC of 0.630 (95% CI 0.628–0.651) [8].

One plausible reason that polygenic risk scores do not substantially increase the AUC for breast cancer prediction models is that these polygenic risk scores only consider linear combinations of SNPs [9]. Detailed models of interactions within a cell reveal complex pathways with many redundancies. Hence, genetic risk for breast cancers might entail non-linear interactions between different genetic factors. Modern machine learning algorithms allow one to consider the effects of non-linear combinations in a model. However, these machine learning algorithms require many more samples (patients) than features (SNPs).

We have introduced a new method of computing genetic risk scores based on chromosomal scale length variation [10–13]. Chromosomal scale length variation characterizes each person with a series of numbers. Each number represents the “length” of a germline chromosome. The “length” is computed from copy number variation measurements made at SNP locations. This “length” varies from person to person because of chromosomal rearrangements: insertions, deletions, translocations, and duplications. These chromosomal rearrangement values are combined across each chromosome (or fractions of a chromosome) to provide a measure of “length.”

After characterizing each person with a series of number derived from their chromosomal scale length variation, we can use the power of modern machine learning algorithms to identify patterns in the germline genetics. The purpose of this paper is to evaluate how well, measured by the AUC, that chromosomal scale length variation can predict breast cancer in patients.

## Methods

To test how well chromosomal length variation can predict breast cancer we acquired germline genetic data on breast cancer patients and non-breast cancer patients (for a control group) from two different data sources, the Cancer Genome Atlas (TCGA) [11, 14, 15] and the UK Biobank [16] project.

The Cancer Genome Atlas (TCGA) characterized molecular differences in 33 different human cancers [14, 15]. The project collected samples from about 11,000 different patients. The project collected multiple samples from each patient, including tissue samples of the tumor and normal tissue adjacent to the tumor and normal blood samples.

Each patient’s germline DNA was extracted from the normal blood samples. A single laboratory processed all germline DNA samples. Each patient’s germline DNA was genotyped by single nucleotide polymorphisms (SNPs) using an Affymetrix SNP 6.0 array. This SNP data were then processed (by the TCGA project) through a bioinformatics pipeline, which included the packages Birdsuite [17] and DNAcopy [18]. The pipeline produced a listing of a chromosomal regions (characterized by the chromosome number, a starting location, and an ending location) and an associated value given as the “segmented mean value” for each patient. The segmented mean value is defined as the logarithm, base 2, of one-half the copy number. A normal diploid region with two copies will have a segmented mean value of zero.

The Genomic Data Commons, an NCI sponsored data repository, contains most of the TCGA data [19]. In the Genomic Data Commons, the copy number variation data is called the masked copy number variation. The masking process removes “Y chromosome and probe sets that were previously indicated to have frequent germline copy-number variation.”

We refer to the final TCGA dataset we used as the masked copy number variation dataset. This dataset originates from normal blood samples extracted from 8826 different patients: 4692 females and 4134 males. The patients’ ages ranged from 10 to 90 years old.

This dataset contains about 695,000 different copy number variations that appear in at least one patient. Copy number variations are genomic regions characterized by the chromosome number, a start position, an end position, and a copy number value. The copy number value is represented as the log base 2 of the ratio of copies present to the expected number of copies, two. A “0” would represent the expected number of copies log2(1), a negative number indicates deletions, and a positive number indicates multiple copies. In the TCGA dataset, normal regions, those with a log_2 CNV equal to 0 are not recorded.

While most copy number variations output by this TCGA pipeline are relatively short, less than the size of a gene, we noted that a few are relatively long, consisting of most of the chromosome’s entire length. For instance, the copy number variation output by the TCGA pipeline that we use to characterize chromosome 1 is 244 megabases long, while the full length of chromosome 1 is 249 megabases. This process produced a dataset with 8,826 rows (each representing a different patient) and 23 columns (each representing one of the chromosomes 1–22 and the X chromosome).

We created a case/control study to differentiate between people with breast cancer and those without breast cancer. For the case/control study, we included all cases in the TCGA dataset that included “normal blood” samples from women patients with a breast cancer diagnosis. No cases were excluded. Patients included also have measurements of copy number variation from DNA derived from normal blood in the database.

Controls for the TCGA dataset include all women in the dataset who had “normal blood” samples without a breast cancer diagnosis. We included only women (no men) in the control sample. Due to the nature of TCGA, each woman in the “control” sample had another type of cancer diagnosis (not breast cancer). The final dataset included 874 women with breast cancer and 3381 women as controls.

To ensure that the results from the TCGA dataset were not due to systematic effects in producing the data, we tested the same methods in a second independent dataset, the UK Biobank. Because of differences in the way in which the data was made available, we could not directly test the predictive power of a model developed on TCGA data with UK Biobank data and vice versa.

We obtained data from the UK Biobank under Application Number 47850. The UK Biobank project collected genetic data and medical records from about 500,000 people who were between the ages of 40 and 69 during the 2006–2010 recruitment years. Most have supplied biological samples and filled out questionnaires about their health. Most of the participants’ medical records are linked, through the National Health Service, to the UK Biobank records. This linkage provides for ongoing follow-up of health conditions [20, 21].

As previously described [12], we first downloaded the “l2r” files from the UK Biobank (version 1). Each chromosome has a separate “l2r” file. Each “l2r” file contained 488,377 columns and a variable number of rows. Each column represented a unique patient in the dataset, who can be identified with an encoded ID number. Each row represented a different location in the genome. The values in the file represent the log base 2 ratio of intensity relative to the expected two copies measured at the SNP location.

We next computed the mean l2r value for different portions of each chromosome for each patient in the dataset, which we refer to as the “length”. We compute the length for each person’s chromosome using the l2r files by taking the average of all l2r values measured across that chromosome. A value of 0 represents the nominal average length of that portion of the chromosome. We call this dataset the chromosome-scale length variation (CSLV) dataset.

This CSLV dataset was joined with the UK Biobank Health records dataset. UK Biobank matched the person in the Public Health England data with UK Biobanks internal records to produce the person’s encoded participant ID. The dataset we have, provided by UK Biobank, contains the participant ID and whether the participant had been diagnosed with breast cancer.

The UK Biobank dataset that we used consisted of measurements at 820,967 genetic markers across 23 chromosomes for each of 488,377 different patients. From the UK Biobank population, we constructed a dataset of positive cases that included all women who both self-reported having been diagnosed with breast cancer and were identified by cancer registries as having been diagnosed with breast cancer, a total of 1534 women. We then constructed a control dataset from a pool of 10,000 UK Biobank participants. From this pool of 10,000 we excluded all men and any women that had any type of cancer diagnosis, either self-reported or from a cancer registry. This gave a control group of 4391 cancer-free.

We quantified the germ line DNA of each of these women with 88 numbers, each representing the length variation of one quarter of each of 22 chromosomes. We did not use the X chromosome.

For both the TCGA data and the UK Biobank data, we set up as a binary classification supervised learning task that was trying to distinguish between patients diagnosed with breast cancer from those not diagnosed with breast cancer.

We performed the analysis using the statistical language R. The data were reformatted for analysis, and then was fed into the machine-learning algorithm. The data included whether the subject had breast cancer, and measurements of copy number variation at distinct locations across the genome derived from the patient’s peripheral blood sample. The data fed into the machine-learning algorithm did not include age since germline DNA should not depend upon age. The results are independent of the patient’s age.

We used the H2O package in R for machine learning. This package implements several common machine learning algorithms, including gradient boosting machines, deep learning neural networks, distributed random forests, and generalized linear models. The best performing algorithms for our datasets are invariably stacked ensembles, which are combinations of other machine learning algorithms. This combination often provides superior results to any particular algorithm [22, 23]. The H2O package implements stacked ensembles as super learner algorithms [24].

*Machine learning* We used the H2O Automatic Machine Learning (automl) function to identify a good machine learning model. The automl function is given a specific amount of time and then proceeds to train and tune a series of models, searching for the best model. To obtain confidence intervals, we repeated the training multiple times (at least 10) with different initial randomization. This process provides independent test/train splits to the data.

The TCGA dataset we used included 874 women with breast cancer and 3381 women who did not have breast cancer as controls. We used 10 × cross validation, so the test set had 87 women with breast cancer and 338 women as controls.

Statistical tests were performed in R. We computed the 95% confidence intervals using the R command t.test. Normality was first confirmed with the Shapiro test. More information is available in Additional file 1.

## Results

**Fig. 1:**
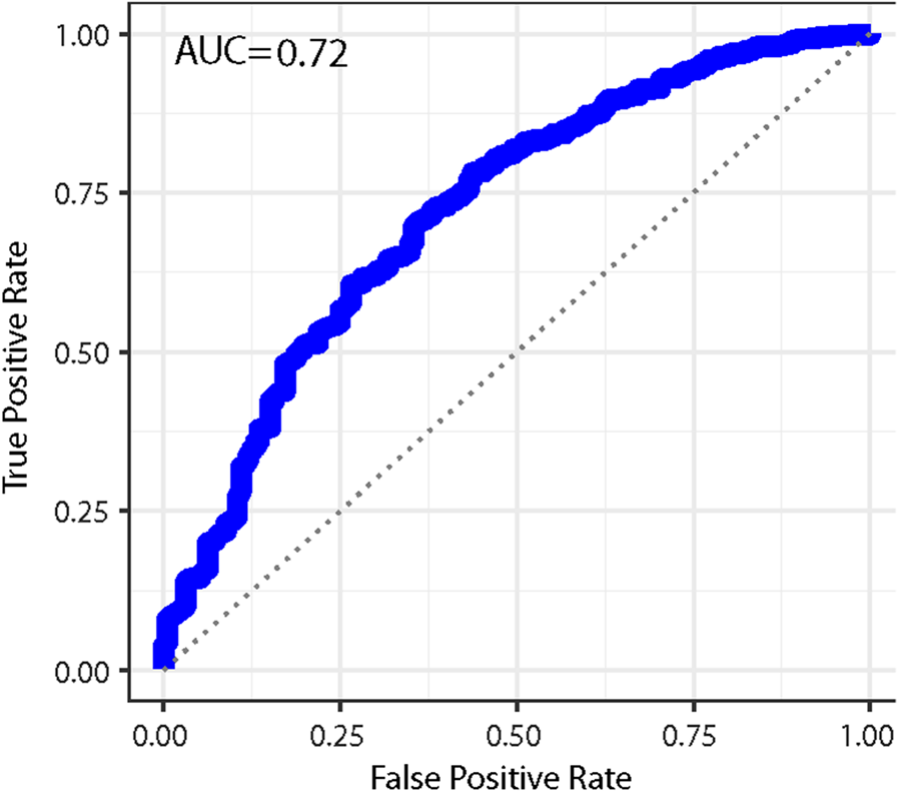
We identified 874 women in the TCGA dataset with breast cancer and 3381 women as controls, women who had another form of cancer but not breast cancer. We characterized the germ line genetics of each of these women with 22 numbers, each one representing the average copy number of a chromosome, or the “length”. Based on this genetic characterization, we found a machine learning algorithm that can classify women with breast cancer compared to other women in the TCGA dataset with an area under the curve of (AUC) of 0.72. This figure depicts the receiver operator characteristic curve

Using the TCGA dataset, consisting of 4,255 women (874 with breast cancer and 3381 controls), we found a classifier with an AUC of 0.704 CI (0.702–0.706) for identifying breast cancer, see Fig. 1. The best classifier identified with the H2O automl package was the gradient boosting machine (GBM) for the TCGA data. We varied the time that automl was allowed to search for better models from one hour to ten hours, but the AUC of the best classifiers were essentially the same, for this range of training times.

**Fig. 2:**
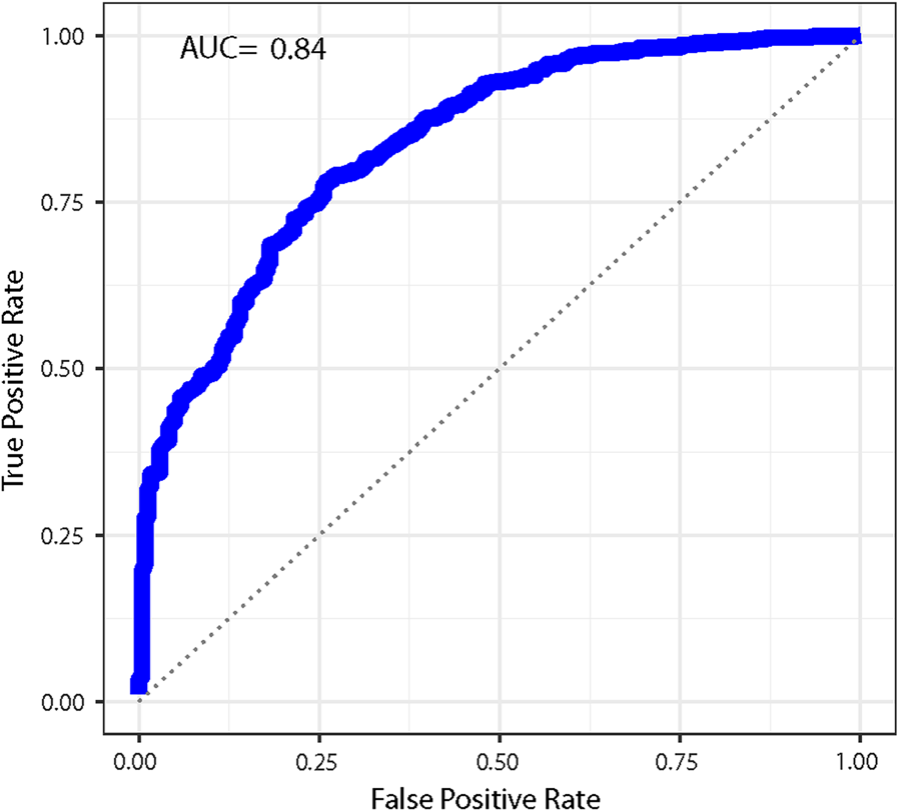
The receiver operator characteristic curves for predicting breast cancer using chromosomal scale length variation with machine learning algorithms. We used a subset of the UK Biobank dataset consisting of 5925 women (1534 who had been diagnosed with breast cancer and 4391 who had never been diagnosed with any form of cancer). We partitioned this group into a training and test set. We used the training set to train algorithms to recognize differences in chromosomal scale length variation data between the women with breast cancer and those without. We then tested this algorithm on the test set. We repeated this process multiple times with different training/test set partitions and found that the AUC was 0.836 with a 95% confidence interval of 0.830 to 0.843

Using our subset of the UK Biobank dataset, consisting of 5925 women (1534 with breast cancer and 4391 normal) with 88 measurements for each, we found a classifier with an AUC of 0.836 with a 95% CI (0.830, 0.843) for identifying breast cancer, see Fig. 2. In this case, the best classifier was a deep learning network, which H2O describes as a multi-layer feedforward artificial neural network trained with stochastic gradient descent. Using the H2O automl function with a time of just one hour, the best individual model was a GBM model, which had an AUC of 0.69 (and a stacked ensemble model with an AUC of 0.76). By increasing the time provided to the automl function to 24 h, it identified a deep learning model with an AUC of 0.81 (and a stacked ensemble model with an AUC of 0.836).

To simulate a real-world application, we then split the UK Biobank dataset into a training set and a test set. The test set consisted of 889 women (233 with breast cancer and 656 without). After obtaining a model from the training set, we applied the model to a test set. The model returns a score for each woman in the test set. The higher the score, the more likely the woman is to have breast cancer. We ranked each woman by the assigned score and then evaluated how accurate the model was for each decile. For instance, about 25% of the women in the test set had breast cancer, but 85% of women who scored in the highest decile had breast cancer. See Table 1 for the detailed results.

**Table 1:** We trained a model to predict breast cancer diagnosis on some UK Biobank data, then tested it on this dataset, which was withheld from the training

Decile	Number of cancers	Number normal	Odds ratio	95% CI
1	75	13	16.8	9.3–30.3
2	57	32	5.2	3.3–8.2
3	36	53	2.0	1.3–3.1
4	15	74	0.59	0.3–1.0
5	15	74	0.59	0.3–1.0
6	10	79	0.37	0.2–0.7
7	5	84	0.17	0.1–0.4
8	9	80	0.33	0.20–0.60
9	2	87	0.07	0.02–0.20
10	3	86	0.10	0.04–0.3
Total	227	662		

This dataset contained 227 patients diagnosed with breast cancer and 662 who had not been diagnosed with breast cancer. The model scored each patient on the likelihood of being classified as breast cancer. The 889 patients were ranked based on their score and split into ten deciles. This table summarized each decile. Those patients who scored in the top decile were 16.8 (95% CI 9.3–30.3) times more likely to have breast cancer than the average woman

**Fig. 3:**
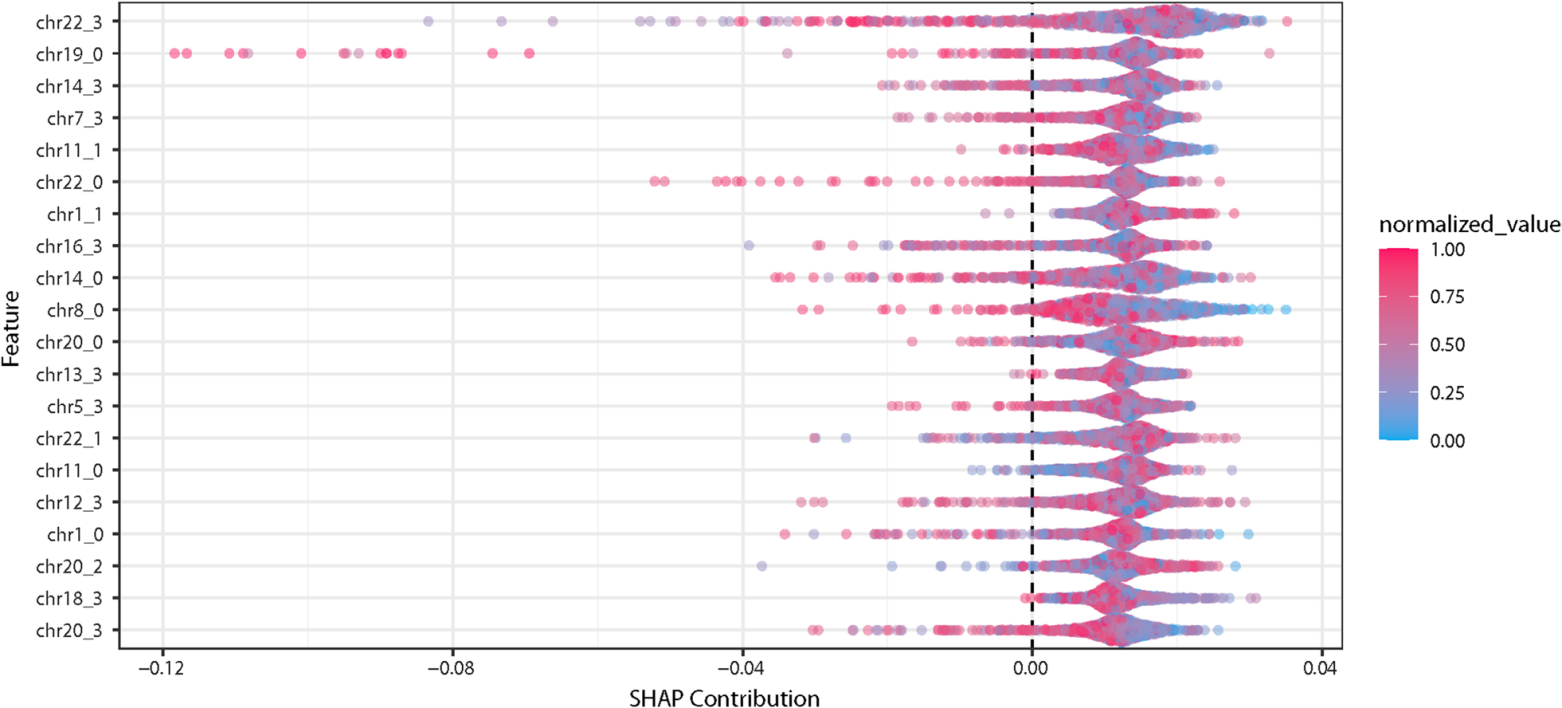
This Shapley additive explanations plot (known as a SHAP plot) provides interpretability to the machine learning model. This SHAP plot is from the UK Biobank machine learning model, shown in Fig. 1. In this model, we used the chromosome-scale length variation on four segments from each chromosome, numbered from 0 to 3. The normalized value represents the value of the parameters. For instance, the red points (closer to 1.0) represent the people with the “longest” associated chromosome, while the blue points (closer to 0) represent people with the shortest associated chromosome. This SHAP plot indicates that the top contribution to the model is from Chromosome 22, segment 3 (the top label on the left axis). However, the SHAP contribution plot also indicates that many different chromosomal regions contribute equally to the model. No one segment is responsible for a majority of the predictive value of the model. Thus, one should not ascribe any particular significance to the third segment of Chromosome 22

We quantified the importance of the different variables (regions of chromosomes) using the summary plot of different SHAP (Shapley Additive exPlanations) contributions. This summary plot assigns each variable an importance for different predictions [25]. The SHAP contribution summary plot for a GBM model on the UK Biobank data is shown in Fig. 3. The algorithm used to generate the SHAP summary plot, TreeSHAP [26], requires a tree based model. Figure 3 is based on the best performing GBM model generated.

## Discussion

Genetic risk scores have been developed for many different ailments [27–30], including cardiovascular disease [27, 31, 32] and different forms of cancer [8, 11, 33, 34].

Breast cancer was the first target of genetic risk scores with the discovery of BRCA1 [35]. A 2015 study that computed a polygenic risk score based on 77 SNPs found that women who scored in the top 1% had a three-fold increase in risk compared to a woman who scored in the middle quintile [36]. For comparison, in the breast cancer prediction presented based on UK Biobank data, here 8 of 9 in the top 1% had breast cancer, while 5 of 27 in the middle 3% had breast cancer.

Machine learning techniques have been used to build polygenic risk scores to predict other complex traits [37, 38]. For instance, diabetes can be predicted from SNP data with an area under the receiver operator curve (AUC) of 0.602 using a gradient boosted regression tree [37].

We evaluated different machine learning algorithms. We used the h2o platform for machine learning and selected the best algorithm by weighing computation time and AUC. The h2o platform evaluated four different algorithms (generalized linear model, distributed random forest, gradient boosting machine, and deeplearning). For the TCGA data, the gradient boosting machine algorithm and the deep learning algorithm provided comparable AUCs, but the gradient boosting algorithm was faster. For the UKBiobank data, the deep learning algorithm provided larger AUCs than all other algorithms evaluated.

Several factors could contribute to the difference between the results we see in TCGA compared to the UK Biobank. The TCGA dataset includes only cancer patients, so the normal people in our TCGA analysis of breast cancer are women who do not have breast cancer but do have some other type of cancer. The TCGA dataset is also more controlled. Each breast tumor in the TCGA study was confirmed by a pathologist to be invasive and either ductal or lobular. The UK Biobank relies more on medical records indicating a diagnosis of “breast cancer.” This diagnosis may include a number of different conditions. Second, the genetic characterization data we have from the UK Biobank is much more extensive than the data from TCGA. The TCGA dataset we constructed relied on the TCGA bioinformatics pipeline, which did not always report a number for factors we were interested in. Many patients had no values reported for a specific chromosome “length,” presumably because it was close normal length and it was not considered a copy number variation. Since we had access to the UK Biobank SP array measurements, we could compute a value for every patient. Third, the UK Biobank population is much more uniform than the TCGA population. The TCGA patients were selected to represent a diverse population. About 76% of the TCGA breast cancer patients were identified as “white”, while about 95% of the UK Biobank participants are categorized as “white.”

The tests have not been optimized. The UKBiobank test could be further improved in two ways. First, the results might improve with further training. The training was done with a desktop computer (Intel i7-3770 with 4 cores, no GPU). We believe additional training time could slightly improve the AUC. Second, we used 88 numbers to characterize each genome, splitting the 22 chromosomes into four equal parts. Some of these regions were highly correlated to others. We might be able to further improve the AUC by splitting the chromosomes into finer parts and ignoring those parts that are highly correlated to existing parts of the dataset.

Understanding the predictions made here is difficult. Although risk prediction and association studies share common methods, the end goals differ. Association studies often try to identify alterations in specific genes that can be mechanistically tied to specific diseases. Risk prediction, however, is only concerned with maximizing the predictive power (18). One method of understanding machine learning models is through examination of variable importance, identifying which regions contribute the most to the model’s predictions. Figure 3 shows the SHAP plot for a predictive model for breast cancer. The figure reveals that no single chromosomal region contributes significantly more than the others to the model’s predictions. The predictions are based on combinations of changes throughout the genome.

We considered whether the results were due to two common problems faced by GWAS studies: batch effects [39] or population stratification. To rule out batch effects, we replicated the results in two independent datasets, the TCGA dataset and the UKBiobank dataset.

Population stratification occurs in case/control studies when the cases and controls contain different proportions of genetically discernable subclasses. Most TCGA samples were collected in the United States from a racially diverse group. The typical process to correct for population stratification in GWAS is to use principal component analysis, but that process in inherently linear and cannot be used with non-linear machine learning techniques.

Cancer is the result of a complex interaction between genetics and the environment. In some cases, for instance, lung cancer and smoking or mesothelioma and asbestos exposure, the required environmental exposure is significant and well known. In other cases, the required environmental exposure is minor and not well known. The genetic signature identified here is a necessary, but not sufficient factor in developing the cancer. Since this is a retrospective study of people who already developed cancer, sufficient environmental exposure has already occurred. A prospective study would need to be performed to determine the effect of environmental exposure on how effective these predictions are.

## Conclusion

In this retrospective study, chromosomal-scale length variation could effectively predict whether or not a woman enrolled in the UK Biobank study developed breast cancer.

## Supplementary Information

**Additional file 1**. Supplemental Methods and Results.

## Data Availability

The datasets analyzed during the current study are available from UK Biobank at https://www.ukbiobank.ac.uk/

## Abbreviations

AUC: Area under the curve
CNV: Copy number variation
CSLV: Chromosomal-scale length variation
GBM: Gradient boosted machines
ROC: Receiver operator curve
SNP: Single nucleotide polymorphism
