# Supplementary material for: Evaluation of a genetic risk score computed using human chromosomal-scale length variation to predict breast cancer
Source: Hum Genomics. 2023 Jun 16;17:53. doi: 10.1186/s40246-023-00482-8 (PMC10273758; doi:10.1186/s40246-023-00482-8)
Supplement: Supplementary file 1 — Additional file 1. Supplemental Methods and Results. [file 40246_2023_482_MOESM1_ESM.docx]

**Supplementary Methods:**

For the TCGA data, we looked at the set of Copy Number Variations produced by the TCGA pipeline and selected the longest one for each chromosome. We used the copy published on Google Big Query. The Copy Number Variation dataset includes a list of 695,000 variations identified. Each item in the list includes the patient in which it originated, the chromosome along with the start and end point on the chromosome and finally the “segment mean,” which is the log base 2 of the number of copies of that chromosome.

We noted that the output of the TCGA pipeline reported a segment mean for very long (>95%) of each chromosome. We produced a dataset that contained 23 numbers for each TCGA patient. The 23 numbers were those reported by the TCGA pipeline as the “segment mean” for the largest segment in each chromosome.

For the UK Biobank data, we had access to the transformed intensity values from the SNP arrays. [The UK Biobank calls this the CNV log2ratios file](https://biobank.ndph.ox.ac.uk/ukb/label.cgi?id=100315). There are separate files for each chromosome. These files contain a list of transformed intensity values measured for each person at each of the specified SNP locations on a given chromosome. To produce the UK Biobank dataset, we computed the average of the transformed intensity for the first 25% of the listed SNP locations, the second 25% of the listed SNP locations, etc. for each chromosome. The UK Biobank dataset contained 88 numbers for each person, since we used 4 numbers from each of their 22 chromosomes. We didn’t use the X and/or Y chromosomes.

We gauged overfitting by performing a “negative control.” The negative control was constructed by taking our actual data and scrambling the breast cancer/control status for each person. The negative control then had the exact same set of patient data, but the breast cancer status had no relation to the patient data. We ran this negative control through our machine learning process and measured the AUC. The measured AUC was always within 0.01 of 0.50, as one would expect for random measurements. Based on this negative control experiment, we do not believe over fitting was a significant factor.

**Code is available at https://github.com/jpbrody/cancer-prediction-cnv**

**Supplementary Table 1. Automl output for UKBiobank.**

This table shows the automl “scoreboard” output for training models on the UK Biobank data. The best models were Stacked Ensembles, which are combinations of more conventional model algorithms. The automl identified a number of deep learning-based models that had AUCs up to 0.827. The best non-deep learning models were a DRF (distributed random forest) model at AUC=0.696 and a GBM (gradient boosting machine) model at AUC=0.695. The best linear model was a GLM (generalized linear model) at AUC=0.595.

|  | model_id | auc | logloss | aucpr | mean_per_class_error | rmse | mse |
| --- | --- | --- | --- | --- | --- | --- | --- |
| 1 | StackedEnsemble_AllModels_4_AutoML_1_20230213_122236 | 0.845 | 0.411 | 0.927 | 0.296 | 0.358 | 0.128 |
| 2 | StackedEnsemble_AllModels_5_AutoML_1_20230213_122236 | 0.844 | 0.407 | 0.924 | 0.274 | 0.357 | 0.128 |
| 3 | StackedEnsemble_AllModels_6_AutoML_1_20230213_122236 | 0.844 | 0.411 | 0.926 | 0.300 | 0.358 | 0.128 |
| 4 | StackedEnsemble_AllModels_3_AutoML_1_20230213_122236 | 0.843 | 0.412 | 0.926 | 0.305 | 0.358 | 0.128 |
| 5 | StackedEnsemble_Best1000_1_AutoML_1_20230213_122236 | 0.843 | 0.413 | 0.926 | 0.297 | 0.359 | 0.129 |
| 6 | DeepLearning_grid_1_AutoML_1_20230213_122236_model_60 | 0.827 | 0.447 | 0.918 | 0.309 | 0.374 | 0.140 |
| 7 | StackedEnsemble_BestOfFamily_6_AutoML_1_20230213_122236 | 0.827 | 0.437 | 0.917 | 0.303 | 0.369 | 0.136 |
| 8 | StackedEnsemble_BestOfFamily_4_AutoML_1_20230213_122236 | 0.826 | 0.437 | 0.918 | 0.312 | 0.369 | 0.136 |
| 9 | DeepLearning_grid_1_AutoML_1_20230213_122236_model_16 | 0.822 | 0.436 | 0.918 | 0.330 | 0.370 | 0.137 |
| 10 | DeepLearning_grid_1_AutoML_1_20230213_122236_model_27 | 0.819 | 0.439 | 0.913 | 0.323 | 0.372 | 0.138 |
| 11 | DeepLearning_grid_1_AutoML_1_20230213_122236_model_18 | 0.819 | 0.467 | 0.910 | 0.314 | 0.376 | 0.141 |
| 12 | StackedEnsemble_BestOfFamily_5_AutoML_1_20230213_122236 | 0.817 | 0.435 | 0.913 | 0.321 | 0.372 | 0.138 |
| 13 | DeepLearning_grid_1_AutoML_1_20230213_122236_model_87 | 0.816 | 0.457 | 0.908 | 0.322 | 0.375 | 0.141 |
| 14 | DeepLearning_grid_1_AutoML_1_20230213_122236_model_1 | 0.812 | 0.458 | 0.908 | 0.327 | 0.380 | 0.144 |
| 15 | DeepLearning_grid_1_AutoML_1_20230213_122236_model_92 | 0.811 | 0.453 | 0.910 | 0.321 | 0.381 | 0.145 |
| 16 | DeepLearning_grid_1_AutoML_1_20230213_122236_model_85 | 0.811 | 0.447 | 0.908 | 0.322 | 0.377 | 0.142 |
| 17 | DeepLearning_grid_1_AutoML_1_20230213_122236_model_49 | 0.805 | 0.463 | 0.906 | 0.341 | 0.384 | 0.148 |
| 18 | DeepLearning_grid_1_AutoML_1_20230213_122236_model_125 | 0.804 | 0.460 | 0.905 | 0.343 | 0.382 | 0.146 |
| 19 | DeepLearning_grid_1_AutoML_1_20230213_122236_model_55 | 0.803 | 0.461 | 0.902 | 0.365 | 0.382 | 0.146 |
| 20 | DeepLearning_grid_1_AutoML_1_20230213_122236_model_82 | 0.802 | 0.456 | 0.905 | 0.329 | 0.382 | 0.146 |
| 21 | DeepLearning_grid_1_AutoML_1_20230213_122236_model_106 | 0.800 | 0.489 | 0.901 | 0.347 | 0.383 | 0.146 |
| 22 | DeepLearning_grid_1_AutoML_1_20230213_122236_model_112 | 0.800 | 0.467 | 0.901 | 0.353 | 0.386 | 0.149 |
| 23 | DeepLearning_grid_1_AutoML_1_20230213_122236_model_40 | 0.799 | 0.481 | 0.902 | 0.346 | 0.382 | 0.146 |
| 24 | DeepLearning_grid_1_AutoML_1_20230213_122236_model_101 | 0.799 | 0.455 | 0.902 | 0.355 | 0.380 | 0.145 |
| 25 | DeepLearning_grid_1_AutoML_1_20230213_122236_model_74 | 0.799 | 0.473 | 0.904 | 0.353 | 0.382 | 0.146 |
| 26 | DeepLearning_grid_1_AutoML_1_20230213_122236_model_126 | 0.798 | 0.465 | 0.906 | 0.347 | 0.387 | 0.149 |
| 27 | DeepLearning_grid_1_AutoML_1_20230213_122236_model_79 | 0.798 | 0.456 | 0.900 | 0.346 | 0.380 | 0.144 |
| 28 | DeepLearning_grid_1_AutoML_1_20230213_122236_model_52 | 0.796 | 0.458 | 0.901 | 0.349 | 0.383 | 0.146 |
| 29 | DeepLearning_grid_1_AutoML_1_20230213_122236_model_28 | 0.795 | 0.464 | 0.899 | 0.346 | 0.384 | 0.148 |
| 30 | DeepLearning_grid_1_AutoML_1_20230213_122236_model_9 | 0.795 | 0.483 | 0.901 | 0.334 | 0.385 | 0.148 |
| 31 | DeepLearning_grid_1_AutoML_1_20230213_122236_model_32 | 0.794 | 0.476 | 0.898 | 0.359 | 0.386 | 0.149 |
| 32 | DeepLearning_grid_1_AutoML_1_20230213_122236_model_71 | 0.793 | 0.468 | 0.901 | 0.388 | 0.387 | 0.150 |
| 33 | DeepLearning_grid_1_AutoML_1_20230213_122236_model_70 | 0.792 | 0.479 | 0.900 | 0.375 | 0.386 | 0.149 |
| 34 | DeepLearning_grid_1_AutoML_1_20230213_122236_model_98 | 0.792 | 0.470 | 0.896 | 0.337 | 0.387 | 0.150 |
| 35 | DeepLearning_grid_2_AutoML_1_20230213_122236_model_18 | 0.791 | 0.468 | 0.901 | 0.381 | 0.386 | 0.149 |
| 36 | DeepLearning_grid_2_AutoML_1_20230213_122236_model_23 | 0.790 | 0.470 | 0.898 | 0.391 | 0.387 | 0.150 |
| 37 | DeepLearning_grid_1_AutoML_1_20230213_122236_model_25 | 0.790 | 0.468 | 0.896 | 0.330 | 0.386 | 0.149 |
| 38 | DeepLearning_grid_1_AutoML_1_20230213_122236_model_88 | 0.790 | 0.463 | 0.896 | 0.338 | 0.384 | 0.148 |
| 39 | DeepLearning_grid_1_AutoML_1_20230213_122236_model_42 | 0.789 | 0.475 | 0.900 | 0.356 | 0.391 | 0.153 |
| 40 | DeepLearning_grid_1_AutoML_1_20230213_122236_model_53 | 0.789 | 0.484 | 0.901 | 0.365 | 0.389 | 0.152 |
| 41 | DeepLearning_grid_1_AutoML_1_20230213_122236_model_54 | 0.788 | 0.469 | 0.896 | 0.361 | 0.388 | 0.151 |
| 42 | DeepLearning_grid_1_AutoML_1_20230213_122236_model_83 | 0.787 | 0.484 | 0.901 | 0.377 | 0.390 | 0.152 |
| 43 | DeepLearning_grid_1_AutoML_1_20230213_122236_model_61 | 0.787 | 0.470 | 0.897 | 0.371 | 0.386 | 0.149 |
| 44 | DeepLearning_grid_1_AutoML_1_20230213_122236_model_34 | 0.786 | 0.485 | 0.897 | 0.346 | 0.390 | 0.152 |
| 45 | DeepLearning_grid_1_AutoML_1_20230213_122236_model_108 | 0.785 | 0.469 | 0.897 | 0.350 | 0.387 | 0.150 |
| 46 | DeepLearning_grid_2_AutoML_1_20230213_122236_model_2 | 0.785 | 0.497 | 0.900 | 0.359 | 0.397 | 0.158 |
| 47 | DeepLearning_grid_1_AutoML_1_20230213_122236_model_122 | 0.783 | 0.500 | 0.892 | 0.345 | 0.389 | 0.151 |
| 48 | DeepLearning_grid_1_AutoML_1_20230213_122236_model_109 | 0.783 | 0.515 | 0.890 | 0.342 | 0.391 | 0.153 |
| 49 | DeepLearning_grid_1_AutoML_1_20230213_122236_model_47 | 0.782 | 0.483 | 0.894 | 0.382 | 0.392 | 0.154 |
| 50 | DeepLearning_grid_1_AutoML_1_20230213_122236_model_3 | 0.782 | 0.535 | 0.891 | 0.339 | 0.391 | 0.153 |
| 51 | DeepLearning_grid_1_AutoML_1_20230213_122236_model_58 | 0.781 | 0.474 | 0.893 | 0.371 | 0.390 | 0.152 |
| 52 | DeepLearning_grid_1_AutoML_1_20230213_122236_model_119 | 0.781 | 0.493 | 0.894 | 0.347 | 0.390 | 0.152 |
| 53 | DeepLearning_grid_1_AutoML_1_20230213_122236_model_67 | 0.780 | 0.481 | 0.890 | 0.384 | 0.391 | 0.153 |
| 54 | DeepLearning_grid_1_AutoML_1_20230213_122236_model_15 | 0.779 | 0.471 | 0.890 | 0.373 | 0.389 | 0.151 |
| 55 | DeepLearning_grid_1_AutoML_1_20230213_122236_model_72 | 0.778 | 0.490 | 0.891 | 0.350 | 0.390 | 0.152 |
| 56 | DeepLearning_grid_1_AutoML_1_20230213_122236_model_110 | 0.777 | 0.476 | 0.890 | 0.363 | 0.391 | 0.153 |
| 57 | DeepLearning_grid_1_AutoML_1_20230213_122236_model_57 | 0.777 | 0.474 | 0.894 | 0.400 | 0.391 | 0.153 |
| 58 | DeepLearning_grid_1_AutoML_1_20230213_122236_model_100 | 0.777 | 0.483 | 0.892 | 0.413 | 0.395 | 0.156 |
| 59 | DeepLearning_grid_1_AutoML_1_20230213_122236_model_78 | 0.777 | 0.491 | 0.890 | 0.353 | 0.393 | 0.155 |
| 60 | DeepLearning_grid_1_AutoML_1_20230213_122236_model_77 | 0.776 | 0.481 | 0.887 | 0.366 | 0.393 | 0.154 |
| 61 | DeepLearning_grid_1_AutoML_1_20230213_122236_model_91 | 0.776 | 0.506 | 0.894 | 0.362 | 0.396 | 0.157 |
| 62 | DeepLearning_grid_1_AutoML_1_20230213_122236_model_90 | 0.775 | 0.482 | 0.890 | 0.407 | 0.394 | 0.155 |
| 63 | DeepLearning_grid_1_AutoML_1_20230213_122236_model_29 | 0.775 | 0.475 | 0.890 | 0.386 | 0.391 | 0.153 |
| 64 | DeepLearning_grid_1_AutoML_1_20230213_122236_model_96 | 0.774 | 0.531 | 0.890 | 0.360 | 0.400 | 0.160 |
| 65 | DeepLearning_grid_1_AutoML_1_20230213_122236_model_84 | 0.774 | 0.487 | 0.891 | 0.374 | 0.395 | 0.156 |
| 66 | DeepLearning_grid_1_AutoML_1_20230213_122236_model_39 | 0.774 | 0.484 | 0.887 | 0.379 | 0.392 | 0.154 |
| 67 | DeepLearning_grid_3_AutoML_1_20230213_122236_model_4 | 0.774 | 0.482 | 0.891 | 0.369 | 0.395 | 0.156 |
| 68 | DeepLearning_grid_1_AutoML_1_20230213_122236_model_94 | 0.773 | 0.484 | 0.883 | 0.343 | 0.391 | 0.153 |
| 69 | DeepLearning_grid_1_AutoML_1_20230213_122236_model_10 | 0.772 | 0.487 | 0.885 | 0.397 | 0.395 | 0.156 |
| 70 | DeepLearning_grid_2_AutoML_1_20230213_122236_model_8 | 0.771 | 0.515 | 0.889 | 0.385 | 0.399 | 0.159 |
| 71 | DeepLearning_grid_1_AutoML_1_20230213_122236_model_120 | 0.770 | 1.754 | 0.885 | 0.343 | 0.446 | 0.199 |
| 72 | DeepLearning_grid_1_AutoML_1_20230213_122236_model_45 | 0.769 | 0.484 | 0.887 | 0.346 | 0.394 | 0.155 |
| 73 | DeepLearning_grid_1_AutoML_1_20230213_122236_model_23 | 0.769 | 0.488 | 0.889 | 0.379 | 0.396 | 0.157 |
| 74 | DeepLearning_grid_1_AutoML_1_20230213_122236_model_8 | 0.768 | 0.487 | 0.886 | 0.402 | 0.396 | 0.157 |
| 75 | DeepLearning_grid_1_AutoML_1_20230213_122236_model_19 | 0.768 | 0.491 | 0.885 | 0.415 | 0.399 | 0.159 |
| 76 | DeepLearning_grid_1_AutoML_1_20230213_122236_model_41 | 0.768 | 0.494 | 0.887 | 0.383 | 0.395 | 0.156 |
| 77 | DeepLearning_grid_2_AutoML_1_20230213_122236_model_29 | 0.768 | 0.494 | 0.887 | 0.385 | 0.403 | 0.162 |
| 78 | DeepLearning_grid_1_AutoML_1_20230213_122236_model_35 | 0.767 | 0.483 | 0.885 | 0.401 | 0.395 | 0.156 |
| 79 | DeepLearning_grid_3_AutoML_1_20230213_122236_model_13 | 0.767 | 0.491 | 0.891 | 0.406 | 0.398 | 0.158 |
| 80 | DeepLearning_grid_1_AutoML_1_20230213_122236_model_44 | 0.767 | 0.493 | 0.882 | 0.379 | 0.394 | 0.155 |
| 81 | DeepLearning_grid_1_AutoML_1_20230213_122236_model_103 | 0.766 | 0.502 | 0.883 | 0.387 | 0.398 | 0.158 |
| 82 | DeepLearning_grid_2_AutoML_1_20230213_122236_model_11 | 0.766 | 0.479 | 0.886 | 0.383 | 0.393 | 0.155 |
| 83 | DeepLearning_grid_3_AutoML_1_20230213_122236_model_12 | 0.766 | 0.488 | 0.888 | 0.426 | 0.399 | 0.159 |
| 84 | DeepLearning_grid_1_AutoML_1_20230213_122236_model_65 | 0.765 | 0.486 | 0.886 | 0.369 | 0.396 | 0.157 |
| 85 | DeepLearning_grid_3_AutoML_1_20230213_122236_model_20 | 0.765 | 0.494 | 0.889 | 0.404 | 0.402 | 0.161 |
| 86 | DeepLearning_grid_1_AutoML_1_20230213_122236_model_81 | 0.763 | 0.500 | 0.886 | 0.383 | 0.400 | 0.160 |
| 87 | DeepLearning_grid_3_AutoML_1_20230213_122236_model_10 | 0.762 | 0.494 | 0.887 | 0.399 | 0.402 | 0.161 |
| 88 | DeepLearning_grid_1_AutoML_1_20230213_122236_model_51 | 0.762 | 0.489 | 0.880 | 0.400 | 0.397 | 0.157 |
| 89 | DeepLearning_grid_1_AutoML_1_20230213_122236_model_4 | 0.760 | 0.514 | 0.883 | 0.415 | 0.401 | 0.161 |
| 90 | DeepLearning_grid_2_AutoML_1_20230213_122236_model_7 | 0.759 | 0.492 | 0.884 | 0.374 | 0.398 | 0.159 |
| 91 | DeepLearning_grid_1_AutoML_1_20230213_122236_model_2 | 0.758 | 0.504 | 0.875 | 0.383 | 0.395 | 0.156 |
| 92 | DeepLearning_grid_2_AutoML_1_20230213_122236_model_14 | 0.757 | 0.489 | 0.880 | 0.395 | 0.399 | 0.159 |
| 93 | DeepLearning_grid_1_AutoML_1_20230213_122236_model_80 | 0.756 | 0.489 | 0.882 | 0.387 | 0.398 | 0.158 |
| 94 | DeepLearning_grid_1_AutoML_1_20230213_122236_model_12 | 0.756 | 0.489 | 0.878 | 0.395 | 0.398 | 0.158 |
| 95 | DeepLearning_grid_1_AutoML_1_20230213_122236_model_13 | 0.756 | 0.509 | 0.877 | 0.386 | 0.401 | 0.161 |
| 96 | DeepLearning_grid_3_AutoML_1_20230213_122236_model_2 | 0.755 | 0.491 | 0.882 | 0.398 | 0.400 | 0.160 |
| 97 | DeepLearning_grid_2_AutoML_1_20230213_122236_model_21 | 0.754 | 0.496 | 0.880 | 0.425 | 0.399 | 0.159 |
| 98 | DeepLearning_grid_2_AutoML_1_20230213_122236_model_13 | 0.751 | 0.498 | 0.883 | 0.444 | 0.404 | 0.163 |
| 99 | DeepLearning_grid_1_AutoML_1_20230213_122236_model_36 | 0.750 | 0.507 | 0.880 | 0.402 | 0.402 | 0.162 |
| 10 | 0 DeepLearning_grid_1_AutoML_1_20230213_122236_model_14 | 0.748 | 0.496 | 0.871 | 0.421 | 0.400 | 0.160 |
| 10 | 1 DeepLearning_grid_1_AutoML_1_20230213_122236_model_117 | 0.748 | 0.509 | 0.872 | 0.396 | 0.400 | 0.160 |
| 10 | 2 DeepLearning_grid_1_AutoML_1_20230213_122236_model_30 | 0.747 | 0.503 | 0.873 | 0.413 | 0.404 | 0.163 |
| 10 | 3 DeepLearning_grid_1_AutoML_1_20230213_122236_model_26 | 0.746 | 0.498 | 0.870 | 0.416 | 0.401 | 0.161 |
| 10 | 4 DeepLearning_grid_1_AutoML_1_20230213_122236_model_115 | 0.745 | 0.502 | 0.873 | 0.410 | 0.404 | 0.163 |
| 10 | 5 DeepLearning_grid_3_AutoML_1_20230213_122236_model_3 | 0.745 | 0.526 | 0.877 | 0.414 | 0.416 | 0.173 |
| 10 | 6 DeepLearning_grid_1_AutoML_1_20230213_122236_model_5 | 0.745 | 0.506 | 0.872 | 0.410 | 0.401 | 0.161 |
| 10 | 7 DeepLearning_grid_1_AutoML_1_20230213_122236_model_20 | 0.741 | 0.496 | 0.872 | 0.416 | 0.402 | 0.162 |
| 10 | 8 DeepLearning_grid_2_AutoML_1_20230213_122236_model_19 | 0.741 | 0.497 | 0.875 | 0.411 | 0.403 | 0.162 |
| 10 | 9 DeepLearning_grid_1_AutoML_1_20230213_122236_model_31 | 0.741 | 0.521 | 0.873 | 0.409 | 0.409 | 0.167 |
| 11 | 0 DeepLearning_grid_1_AutoML_1_20230213_122236_model_123 | 0.739 | 0.501 | 0.871 | 0.395 | 0.403 | 0.163 |
| 11 | 1 DeepLearning_grid_1_AutoML_1_20230213_122236_model_64 | 0.738 | 0.517 | 0.866 | 0.396 | 0.404 | 0.163 |
| 11 | 2 DeepLearning_grid_1_AutoML_1_20230213_122236_model_59 | 0.737 | 0.501 | 0.867 | 0.406 | 0.403 | 0.162 |
| 11 | 3 DeepLearning_grid_3_AutoML_1_20230213_122236_model_9 | 0.737 | 0.505 | 0.869 | 0.420 | 0.405 | 0.164 |
| 11 | 4 DeepLearning_grid_2_AutoML_1_20230213_122236_model_10 | 0.737 | 0.531 | 0.873 | 0.430 | 0.411 | 0.169 |
| 11 | 5 DeepLearning_grid_1_AutoML_1_20230213_122236_model_43 | 0.736 | 0.543 | 0.873 | 0.421 | 0.413 | 0.170 |
| 11 | 6 DeepLearning_grid_1_AutoML_1_20230213_122236_model_104 | 0.734 | 0.517 | 0.869 | 0.426 | 0.407 | 0.166 |
| 11 | 7 DeepLearning_grid_1_AutoML_1_20230213_122236_model_6 | 0.734 | 0.566 | 0.864 | 0.422 | 0.410 | 0.168 |
| 11 | 8 DeepLearning_grid_3_AutoML_1_20230213_122236_model_19 | 0.733 | 0.502 | 0.873 | 0.409 | 0.405 | 0.164 |
| 11 | 9 DeepLearning_grid_3_AutoML_1_20230213_122236_model_21 | 0.733 | 0.503 | 0.873 | 0.443 | 0.406 | 0.165 |
| 12 | 0 DeepLearning_grid_1_AutoML_1_20230213_122236_model_22 | 0.732 | 0.521 | 0.863 | 0.426 | 0.408 | 0.166 |
| 12 | 1 DeepLearning_grid_2_AutoML_1_20230213_122236_model_15 | 0.731 | 0.504 | 0.871 | 0.414 | 0.405 | 0.164 |
| 12 | 2 DeepLearning_grid_3_AutoML_1_20230213_122236_model_22 | 0.731 | 0.504 | 0.870 | 0.428 | 0.406 | 0.165 |
| 12 | 3 DeepLearning_grid_1_AutoML_1_20230213_122236_model_68 | 0.730 | 0.518 | 0.865 | 0.435 | 0.409 | 0.168 |
| 12 | 4 DeepLearning_grid_1_AutoML_1_20230213_122236_model_107 | 0.730 | 0.521 | 0.866 | 0.419 | 0.408 | 0.167 |
| 12 | 5 DeepLearning_grid_1_AutoML_1_20230213_122236_model_46 | 0.729 | 0.546 | 0.859 | 0.425 | 0.410 | 0.168 |
| 12 | 6 DeepLearning_grid_1_AutoML_1_20230213_122236_model_7 | 0.729 | 0.537 | 0.863 | 0.429 | 0.410 | 0.168 |
| 12 | 7 DeepLearning_grid_3_AutoML_1_20230213_122236_model_7 | 0.729 | 0.506 | 0.869 | 0.411 | 0.406 | 0.165 |
| 12 | 8 DeepLearning_grid_1_AutoML_1_20230213_122236_model_69 | 0.729 | 0.510 | 0.865 | 0.427 | 0.406 | 0.165 |
| 12 | 9 DeepLearning_grid_1_AutoML_1_20230213_122236_model_111 | 0.728 | 0.510 | 0.866 | 0.423 | 0.408 | 0.166 |
| 13 | 0 DeepLearning_grid_2_AutoML_1_20230213_122236_model_28 | 0.728 | 0.510 | 0.867 | 0.420 | 0.409 | 0.167 |
| 13 | 1 DeepLearning_grid_1_AutoML_1_20230213_122236_model_75 | 0.727 | 0.508 | 0.865 | 0.432 | 0.407 | 0.166 |
| 13 | 2 DeepLearning_grid_2_AutoML_1_20230213_122236_model_5 | 0.726 | 0.518 | 0.866 | 0.441 | 0.410 | 0.168 |
| 13 | 3 DeepLearning_grid_3_AutoML_1_20230213_122236_model_18 | 0.725 | 0.512 | 0.865 | 0.431 | 0.409 | 0.168 |
| 13 | 4 DeepLearning_grid_1_AutoML_1_20230213_122236_model_56 | 0.723 | 0.539 | 0.861 | 0.408 | 0.416 | 0.173 |
| 13 | 5 DeepLearning_grid_1_AutoML_1_20230213_122236_model_38 | 0.722 | 0.514 | 0.865 | 0.430 | 0.409 | 0.167 |
| 13 | 6 DeepLearning_grid_3_AutoML_1_20230213_122236_model_6 | 0.721 | 0.520 | 0.864 | 0.428 | 0.410 | 0.168 |
| 13 | 7 DeepLearning_grid_3_AutoML_1_20230213_122236_model_16 | 0.720 | 0.525 | 0.865 | 0.430 | 0.411 | 0.169 |
| 13 | 8 DeepLearning_grid_2_AutoML_1_20230213_122236_model_6 | 0.720 | 0.529 | 0.856 | 0.421 | 0.411 | 0.169 |
| 13 | 9 DeepLearning_grid_2_AutoML_1_20230213_122236_model_12 | 0.720 | 0.525 | 0.865 | 0.425 | 0.411 | 0.169 |
| 14 | 0 DeepLearning_grid_1_AutoML_1_20230213_122236_model_37 | 0.719 | 0.559 | 0.850 | 0.384 | 0.420 | 0.176 |
| 14 | 1 DeepLearning_grid_3_AutoML_1_20230213_122236_model_11 | 0.717 | 0.564 | 0.861 | 0.433 | 0.423 | 0.179 |
| 14 | 2 DeepLearning_grid_1_AutoML_1_20230213_122236_model_99 | 0.717 | 0.526 | 0.858 | 0.421 | 0.412 | 0.169 |
| 14 | 3 DeepLearning_grid_3_AutoML_1_20230213_122236_model_5 | 0.716 | 0.547 | 0.860 | 0.410 | 0.419 | 0.176 |
| 14 | 4 DeepLearning_grid_1_AutoML_1_20230213_122236_model_48 | 0.715 | 0.518 | 0.858 | 0.422 | 0.409 | 0.167 |
| 14 | 5 StackedEnsemble_AllModels_2_AutoML_1_20230213_122236 | 0.715 | 0.510 | 0.858 | 0.437 | 0.409 | 0.167 |
| 14 | 6 StackedEnsemble_BestOfFamily_3_AutoML_1_20230213_122236 | 0.714 | 0.510 | 0.858 | 0.436 | 0.409 | 0.167 |
| 14 | 7 DeepLearning_grid_2_AutoML_1_20230213_122236_model_4 | 0.714 | 0.519 | 0.861 | 0.428 | 0.409 | 0.167 |
| 14 | 8 StackedEnsemble_AllModels_1_AutoML_1_20230213_122236 | 0.712 | 0.513 | 0.858 | 0.432 | 0.410 | 0.168 |
| 14 | 9 StackedEnsemble_BestOfFamily_2_AutoML_1_20230213_122236 | 0.711 | 0.513 | 0.858 | 0.437 | 0.410 | 0.168 |
| 15 | 0 DeepLearning_grid_2_AutoML_1_20230213_122236_model_24 | 0.709 | 0.516 | 0.849 | 0.427 | 0.411 | 0.169 |
| 15 | 1 DeepLearning_grid_2_AutoML_1_20230213_122236_model_17 | 0.705 | 0.531 | 0.854 | 0.436 | 0.414 | 0.172 |
| 15 | 2 DeepLearning_grid_1_AutoML_1_20230213_122236_model_124 | 0.705 | 0.539 | 0.845 | 0.409 | 0.413 | 0.170 |
| 15 | 3 DeepLearning_grid_3_AutoML_1_20230213_122236_model_15 | 0.704 | 0.537 | 0.854 | 0.433 | 0.417 | 0.174 |
| 15 | 4 DeepLearning_grid_2_AutoML_1_20230213_122236_model_9 | 0.703 | 0.569 | 0.854 | 0.437 | 0.420 | 0.176 |
| 15 | 5 DeepLearning_grid_1_AutoML_1_20230213_122236_model_105 | 0.701 | 0.550 | 0.845 | 0.436 | 0.419 | 0.175 |
| 15 | 6 DeepLearning_grid_2_AutoML_1_20230213_122236_model_31 | 0.701 | 0.534 | 0.853 | 0.420 | 0.416 | 0.173 |
| 15 | 7 DeepLearning_grid_2_AutoML_1_20230213_122236_model_20 | 0.701 | 0.525 | 0.859 | 0.444 | 0.417 | 0.174 |
| 15 | 8 DeepLearning_grid_3_AutoML_1_20230213_122236_model_17 | 0.701 | 0.546 | 0.853 | 0.440 | 0.420 | 0.177 |
| 15 | 9 DeepLearning_grid_1_AutoML_1_20230213_122236_model_11 | 0.700 | 0.544 | 0.843 | 0.441 | 0.417 | 0.174 |
| 16 | 0 DeepLearning_grid_1_AutoML_1_20230213_122236_model_97 | 0.700 | 0.551 | 0.841 | 0.422 | 0.414 | 0.171 |
| 16 | 1 DeepLearning_grid_1_AutoML_1_20230213_122236_model_21 | 0.698 | 0.525 | 0.840 | 0.418 | 0.410 | 0.168 |
| 16 | 2 DRF_1_AutoML_1_20230213_122236 | 0.696 | 0.520 | 0.845 | 0.429 | 0.413 | 0.171 |
| 16 | 3 GBM_lr_annealing_selection_AutoML_1_20230213_122236_select_model | 0.695 | 0.519 | 0.841 | 0.443 | 0.413 | 0.170 |
| 16 | 4 StackedEnsemble_BestOfFamily_1_AutoML_1_20230213_122236 | 0.695 | 0.525 | 0.844 | 0.448 | 0.416 | 0.173 |
| 16 | 5 DeepLearning_grid_1_AutoML_1_20230213_122236_model_62 | 0.693 | 0.529 | 0.834 | 0.429 | 0.412 | 0.170 |
| 16 | 6 GBM_grid_1_AutoML_1_20230213_122236_model_2 | 0.692 | 0.522 | 0.839 | 0.433 | 0.414 | 0.171 |
| 16 | 7 GBM_grid_1_AutoML_1_20230213_122236_model_13 | 0.690 | 0.524 | 0.836 | 0.444 | 0.415 | 0.172 |
| 16 | 8 GBM_grid_1_AutoML_1_20230213_122236_model_3 | 0.689 | 0.522 | 0.843 | 0.440 | 0.414 | 0.172 |
| 16 | 9 GBM_4_AutoML_1_20230213_122236 | 0.689 | 0.524 | 0.838 | 0.426 | 0.415 | 0.172 |
| 17 | 0 GBM_grid_1_AutoML_1_20230213_122236_model_6 | 0.689 | 0.522 | 0.839 | 0.430 | 0.414 | 0.172 |
| 17 | 1 GBM_1_AutoML_1_20230213_122236 | 0.688 | 0.526 | 0.840 | 0.447 | 0.416 | 0.173 |
| 17 | 2 DeepLearning_grid_2_AutoML_1_20230213_122236_model_25 | 0.688 | 0.531 | 0.841 | 0.422 | 0.416 | 0.173 |
| 17 | 3 DeepLearning_grid_1_AutoML_1_20230213_122236_model_113 | 0.688 | 0.576 | 0.841 | 0.454 | 0.427 | 0.183 |
| 17 | 4 XRT_1_AutoML_1_20230213_122236 | 0.687 | 0.522 | 0.841 | 0.437 | 0.414 | 0.171 |
| 17 | 5 GBM_grid_1_AutoML_1_20230213_122236_model_5 | 0.685 | 0.524 | 0.833 | 0.443 | 0.415 | 0.172 |
| 17 | 6 DeepLearning_grid_1_AutoML_1_20230213_122236_model_66 | 0.685 | 0.572 | 0.831 | 0.420 | 0.419 | 0.176 |
| 17 | 7 DeepLearning_grid_2_AutoML_1_20230213_122236_model_1 | 0.685 | 0.539 | 0.849 | 0.463 | 0.421 | 0.178 |
| 17 | 8 GBM_grid_1_AutoML_1_20230213_122236_model_12 | 0.685 | 0.528 | 0.837 | 0.436 | 0.417 | 0.174 |
| 17 | 9 GBM_grid_1_AutoML_1_20230213_122236_model_11 | 0.684 | 0.527 | 0.838 | 0.437 | 0.417 | 0.174 |
| 18 | 0 GBM_grid_1_AutoML_1_20230213_122236_model_7 | 0.684 | 0.531 | 0.839 | 0.443 | 0.417 | 0.174 |
| 18 | 1 GBM_2_AutoML_1_20230213_122236 | 0.684 | 0.523 | 0.836 | 0.434 | 0.414 | 0.172 |
| 18 | 2 DeepLearning_grid_1_AutoML_1_20230213_122236_model_93 | 0.683 | 0.564 | 0.832 | 0.444 | 0.423 | 0.179 |
| 18 | 3 GBM_grid_1_AutoML_1_20230213_122236_model_8 | 0.683 | 0.523 | 0.834 | 0.434 | 0.414 | 0.172 |
| 18 | 4 DeepLearning_grid_1_AutoML_1_20230213_122236_model_50 | 0.682 | 0.604 | 0.845 | 0.415 | 0.427 | 0.182 |
| 18 | 5 GBM_5_AutoML_1_20230213_122236 | 0.681 | 0.524 | 0.830 | 0.437 | 0.415 | 0.172 |
| 18 | 6 DeepLearning_grid_2_AutoML_1_20230213_122236_model_16 | 0.680 | 0.562 | 0.841 | 0.465 | 0.430 | 0.185 |
| 18 | 7 GBM_grid_1_AutoML_1_20230213_122236_model_10 | 0.680 | 0.533 | 0.834 | 0.450 | 0.419 | 0.175 |
| 18 | 8 DeepLearning_grid_1_AutoML_1_20230213_122236_model_118 | 0.679 | 0.544 | 0.826 | 0.413 | 0.416 | 0.173 |
| 18 | 9 GBM_grid_1_AutoML_1_20230213_122236_model_9 | 0.678 | 0.526 | 0.828 | 0.432 | 0.416 | 0.173 |
| 19 | 0 DeepLearning_grid_1_AutoML_1_20230213_122236_model_89 | 0.678 | 0.615 | 0.835 | 0.438 | 0.438 | 0.192 |
| 19 | 1 GBM_grid_1_AutoML_1_20230213_122236_model_4 | 0.676 | 0.526 | 0.825 | 0.426 | 0.416 | 0.173 |
| 19 | 2 GBM_grid_1_AutoML_1_20230213_122236_model_1 | 0.676 | 0.527 | 0.826 | 0.422 | 0.416 | 0.173 |
| 19 | 3 DeepLearning_grid_1_AutoML_1_20230213_122236_model_24 | 0.673 | 0.585 | 0.833 | 0.441 | 0.428 | 0.183 |
| 19 | 4 DeepLearning_grid_3_AutoML_1_20230213_122236_model_14 | 0.672 | 0.583 | 0.835 | 0.466 | 0.430 | 0.185 |
| 19 | 5 DeepLearning_grid_3_AutoML_1_20230213_122236_model_23 | 0.670 | 0.539 | 0.838 | 0.448 | 0.423 | 0.179 |
| 19 | 6 DeepLearning_grid_1_AutoML_1_20230213_122236_model_121 | 0.670 | 0.560 | 0.829 | 0.458 | 0.426 | 0.181 |
| 19 | 7 DeepLearning_grid_1_AutoML_1_20230213_122236_model_76 | 0.670 | 0.578 | 0.833 | 0.458 | 0.431 | 0.185 |
| 19 | 8 DeepLearning_grid_1_AutoML_1_20230213_122236_model_116 | 0.662 | 0.537 | 0.810 | 0.435 | 0.419 | 0.175 |
| 19 | 9 DeepLearning_1_AutoML_1_20230213_122236 | 0.661 | 0.544 | 0.818 | 0.433 | 0.424 | 0.180 |
| 20 | 0 DeepLearning_grid_1_AutoML_1_20230213_122236_model_33 | 0.658 | 0.555 | 0.821 | 0.454 | 0.426 | 0.181 |
| 20 | 1 DeepLearning_grid_2_AutoML_1_20230213_122236_model_32 | 0.650 | 0.565 | 0.821 | 0.482 | 0.431 | 0.186 |
| 20 | 2 DeepLearning_grid_1_AutoML_1_20230213_122236_model_95 | 0.642 | 0.649 | 0.815 | 0.461 | 0.442 | 0.196 |
| 20 | 3 DeepLearning_grid_1_AutoML_1_20230213_122236_model_128 | 0.640 | 0.562 | 0.811 | 0.475 | 0.431 | 0.186 |
| 20 | 4 DeepLearning_grid_1_AutoML_1_20230213_122236_model_102 | 0.639 | 0.583 | 0.799 | 0.483 | 0.436 | 0.190 |
| 20 | 5 DeepLearning_grid_1_AutoML_1_20230213_122236_model_114 | 0.634 | 0.590 | 0.800 | 0.480 | 0.434 | 0.189 |
| 20 | 6 DeepLearning_grid_1_AutoML_1_20230213_122236_model_63 | 0.633 | 0.602 | 0.791 | 0.484 | 0.439 | 0.193 |
| 20 | 7 DeepLearning_grid_1_AutoML_1_20230213_122236_model_73 | 0.631 | 0.570 | 0.801 | 0.463 | 0.436 | 0.190 |
| 20 | 8 DeepLearning_grid_1_AutoML_1_20230213_122236_model_17 | 0.614 | 0.561 | 0.778 | 0.461 | 0.429 | 0.184 |
| 20 | 9 DeepLearning_grid_3_AutoML_1_20230213_122236_model_24 | 0.612 | 0.563 | 0.803 | 0.476 | 0.434 | 0.188 |
| 21 | 0 DeepLearning_grid_3_AutoML_1_20230213_122236_model_1 | 0.610 | 0.653 | 0.796 | 0.476 | 0.449 | 0.202 |
| 21 | 1 DeepLearning_grid_2_AutoML_1_20230213_122236_model_26 | 0.609 | 0.747 | 0.805 | 0.492 | 0.461 | 0.213 |
| 21 | 2 DeepLearning_grid_1_AutoML_1_20230213_122236_model_129 | 0.605 | 0.597 | 0.777 | 0.493 | 0.439 | 0.193 |
| 21 | 3 DeepLearning_grid_1_AutoML_1_20230213_122236_model_86 | 0.602 | 0.585 | 0.786 | 0.477 | 0.439 | 0.193 |
| 21 | 4 DeepLearning_grid_2_AutoML_1_20230213_122236_model_22 | 0.602 | 0.608 | 0.798 | 0.489 | 0.442 | 0.196 |
| 21 | 5 DeepLearning_grid_2_AutoML_1_20230213_122236_model_30 | 0.599 | 0.582 | 0.795 | 0.494 | 0.440 | 0.193 |
| 21 | 6 DeepLearning_grid_2_AutoML_1_20230213_122236_model_27 | 0.597 | 0.589 | 0.795 | 0.487 | 0.441 | 0.194 |
| 21 | 7 GLM_1_AutoML_1_20230213_122236 | 0.595 | 0.566 | 0.778 | 0.494 | 0.435 | 0.189 |
| 21 | 8 DeepLearning_grid_2_AutoML_1_20230213_122236_model_3 | 0.590 | 0.691 | 0.785 | 0.494 | 0.454 | 0.206 |
| 21 | 9 DeepLearning_grid_3_AutoML_1_20230213_122236_model_8 | 0.570 | 0.662 | 0.786 | 0.495 | 0.451 | 0.204 |
